# 2-Amino-5-methyl­pyridinium 3-amino­benzoate

**DOI:** 10.1107/S1600536810005180

**Published:** 2010-02-13

**Authors:** Madhukar Hemamalini, Hoong-Kun Fun

**Affiliations:** a X-ray Crystallography Unit, School of Physics, Universiti Sains Malaysia, 11800 USM, Penang, Malaysia

## Abstract

In the title compound, C_6_H_9_N_2_^+^·C_7_H_6_NO_2_^−^, the H atom of the N—H group and an H atom of the 2-amino group from the cation are involved in inter­molecular N—H⋯O hydrogen bonds with the O atoms of the carboxyl­ate group of the anion, forming an *R*_2_^2^(8) ring motif. These ring motifs are, in turn, connected by further N—H⋯O hydrogen bonds, forming a two-dimensional network. The crystal structure is further stabilized by π⋯π stacking inter­actions involving the benzene and pyridinium rings with a centroid–centroid distance of 3.7594 (8) Å.

## Related literature

For background to the chemistry of substituted pyridines see: Pozharski *et al.* (1997 ▶); Katritzky *et al.* (1996 ▶). For related structures, see: Nahringbauer & Kvick (1977 ▶); Feng *et al.* (2005 ▶); Xuan *et al.* (2003 ▶); Jin *et al.* (2005 ▶). For details of hydrogen bonding, see: Jeffrey & Saenger (1991 ▶); Jeffrey (1997 ▶); Scheiner (1997 ▶). For hydrogen-bond motifs, see: Bernstein *et al.* (1995 ▶). For bond-length data, see: Allen *et al.* (1987 ▶). 
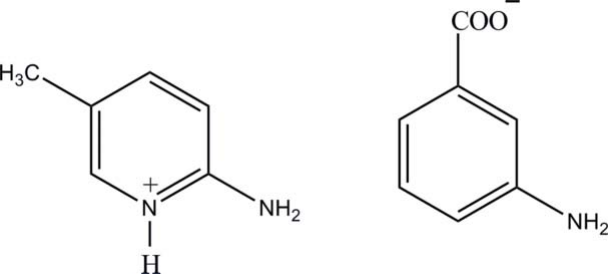


## Experimental

### 

#### Crystal data

C_6_H_9_N_2_^+^·C_7_H_6_NO_2_^−^
*M* *_r_* = 245.28
Monoclinic, 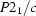

*a* = 10.0739 (2) Å
*b* = 10.9620 (2) Å
*c* = 11.9641 (2) Å
β = 113.148 (1)°
*V* = 1214.83 (4) Å^3^
*Z* = 4
Mo *K*α radiation
μ = 0.09 mm^−1^
*T* = 296 K
0.72 × 0.34 × 0.13 mm

#### Data collection

Bruker SMART APEXII CCD area-detector diffractometer
Absorption correction: multi-scan (*SADABS*; Bruker, 2009 ▶) *T*_min_ = 0.936, *T*_max_ = 0.988
13305 measured reflections
3541 independent reflections
2576 reflections with *I* > 2σ(*I*)
*R*_int_ = 0.029

#### Refinement

*R*[*F* ^2^ > 2σ(*F* ^2^)] = 0.048
*wR*(*F* ^2^) = 0.138
*S* = 1.07
3541 reflections
212 parameters
H atoms treated by a mixture of independent and constrained refinement
Δρ_max_ = 0.20 e Å^−3^
Δρ_min_ = −0.26 e Å^−3^

### 

Data collection: *APEX2* (Bruker, 2009 ▶); cell refinement: *SAINT* (Bruker, 2009 ▶); data reduction: *SAINT*; program(s) used to solve structure: *SHELXTL* (Sheldrick, 2008 ▶); program(s) used to refine structure: *SHELXTL*; molecular graphics: *SHELXTL* software used to prepare material for publication: *SHELXTL* and *PLATON* (Spek, 2009 ▶).

## Supplementary Material

Crystal structure: contains datablocks global, I. DOI: 10.1107/S1600536810005180/lh2994sup1.cif

Structure factors: contains datablocks I. DOI: 10.1107/S1600536810005180/lh2994Isup2.hkl

Additional supplementary materials:  crystallographic information; 3D view; checkCIF report

## Figures and Tables

**Table 1:** Hydrogen-bond geometry (Å, °)

*D*—H⋯*A*	*D*—H	H⋯*A*	*D*⋯*A*	*D*—H⋯*A*
N1—H1*N*1⋯O2^i^	1.017 (17)	1.682 (17)	2.6901 (14)	170.6 (17)
N2—H1*N*2⋯O1^i^	0.939 (16)	1.886 (16)	2.8207 (15)	173.3 (14)
N2—H2*N*2⋯O2^ii^	0.920 (17)	1.947 (17)	2.8650 (16)	175.3 (16)
N3—H1*N*3⋯O1^iii^	0.903 (19)	2.18 (2)	3.027 (2)	156.0 (17)

Symmetry codes: (i) 
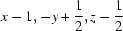
; (ii) 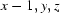; (iii) 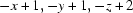.

**Fig. 1.**
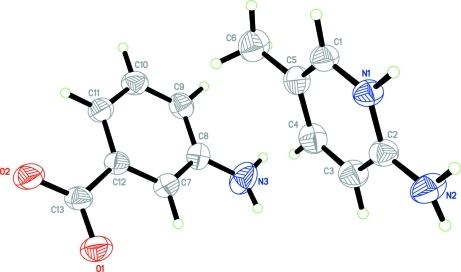
The asymmetric unit of the title compound. Displacement ellipsoids are drawn at the 50% probability level.

**Fig. 2.**
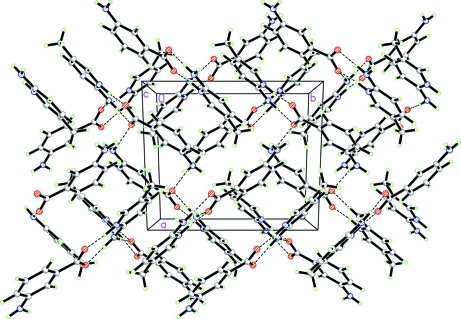
The crystal packing of the title compound, showing hydrogen-bonded (dashed lines) networks.

## supplementary crystallographic information

### Comment

Pyridine and its derivatives play an important role in heterocyclic chemistry (Pozharski *et al.*, 1997; Katritzky *et al.*, 1996). Pyridine and its substituted derivatives are often involved in hydrogen-bond interactions (Jeffrey & Saenger, 1991; Jeffrey, 1997; Scheiner, 1997).  The crystal structures of 2-amino-5-methylpyridine (Nahringbauer & Kvick, 1977), 2-amino-5-methylpyridinium phosphate (Feng *et al.*, 2005), 2-amino-5-methylpyridinium 3-(4- hydroxy-3-methoxyphenyl)-2-propenoate monohydrate (Xuan *et al.*, 2003) and 2-amino-5-methylpyridinium (2-amino-5-methylpyridine)trichlorozincate(II) (Jin *et al.*, 2005) have been reported in the literature. In order to study some interesting hydrogen bonding interactions, the synthesis and structure of the title salt is presented here.

The asymmetric unit (Fig. 1) contains a 2-amino-5-methylpyridinium cation and a 3-aminobenzoate anion. The proton transfer from the carboxyl group to atom N1 of 2-amino-5-methylpyridine resulted in the widening of C2—N1—C1 angle of the pyridinium ring to 122.40 (10)°, compared to the corresponding angle of 117.4° (no standard uncertainty available) in neutral 2-amino-5-methylpyridine (Nahringbauer & Kvick, 1977). The 2-amino-5-methylpyridinium cation is essentially planar,  with a maximum deviation of 0.002 (1)Å for atom N1. The bond lengths (Allen *et al.*, 1987) and angles are within normal ranges.

In the crystal structure (Fig. 2), the protonated N1 atom and 2-amino group (N2) are hydrogen-bonded to the carboxylate oxygen atoms (O1 and O2) via a pair of N—H···O hydrogen bonds forming a ring motif *R*_2_^2^(8) (Bernstein *et al.*, 1995).  The symmetry-related 3-aminobenzoate molecules are linked through N3—H1N3···O1(-x+1, -y+1, -z+2) hydrogen-bonding to form a *R*_2_^2^(14) ring motif (Table 1). The cystal structure is further stabilized by π···π stacking interaction between the pyridine rings (C1–C5/N1) and benzene ring (C7–C12) with centroid- to-centroid distance of 3.7594 (8)Å [symmetry codes: 1-x, 1/2+y, 3/2-z and 1-x, -1/2+y, 3/2-z ].

### Experimental

A hot methanol solution (20 ml) of 2-amino-5-methylpyridine (54 mg, Aldrich) and 3-aminobenzoic acid (68 mg, Merck) were mixed and warmed over a heating magnetic stirrer for a few minutes.  The resulting solution was allowed to cool slowly at room temperature and crystals of the title compound appeared after a few days.

### Refinement

The methyl H atoms were positioned geometrically and were refined using a riding model, with *U*_iso_(H) = 1.5*U*_eq_(C). A rotating group model was used for the methyl group. The remaining H atoms were located in a difference map and refined freely [N–H = 0.92 (2)–1.02 (2)Å, C–H = 0.96–1.00 (2)Å].

### Crystal data

**Table:**

C_6_H_9_N_2_^+^·C_7_H_6_NO_2_^−^	*F*(000) = 520
*M**_r_* = 245.28	*D*_x_ = 1.341 Mg m^−^^3^
Monoclinic, *P*2_1_/*c*	Mo *K*α radiation, λ = 0.71073 Å
Hall symbol: -P 2ybc	Cell parameters from 3778 reflections
*a* = 10.0739 (2) Å	θ = 2.6–29.9°
*b* = 10.9620 (2) Å	µ = 0.09 mm^−^^1^
*c* = 11.9641 (2) Å	*T* = 296 K
β = 113.148 (1)°	Plate, brown
*V* = 1214.83 (4) Å^3^	0.72 × 0.34 × 0.13 mm
*Z* = 4	

### Data collection

**Table:**

Bruker SMART APEXII CCD area-detector diffractometer	3541 independent reflections
Radiation source: fine-focus sealed tube	2576 reflections with *I* > 2σ(*I*)
graphite	*R*_int_ = 0.029
φ and ω scans	θ_max_ = 30.1°, θ_min_ = 2.2°
Absorption correction: multi-scan (*SADABS*; Bruker, 2009)	*h* = −10→14
*T*_min_ = 0.936, *T*_max_ = 0.988	*k* = −15→13
13305 measured reflections	*l* = −16→16

### Refinement

**Table:**

Refinement on *F*^2^	Primary atom site location: structure-invariant direct methods
Least-squares matrix: full	Secondary atom site location: difference Fourier map
*R*[*F*^2^ > 2σ(*F*^2^)] = 0.048	Hydrogen site location: inferred from neighbouring sites
*wR*(*F*^2^) = 0.138	H atoms treated by a mixture of independent and constrained refinement
*S* = 1.07	*w* = 1/[σ^2^(*F*_o_^2^) + (0.0676*P*)^2^ + 0.1203*P*] where *P* = (*F*_o_^2^ + 2*F*_c_^2^)/3
3541 reflections	(Δ/σ)_max_ = 0.001
212 parameters	Δρ_max_ = 0.20 e Å^−^^3^
0 restraints	Δρ_min_ = −0.26 e Å^−^^3^

### Special details

**Table:**

Experimental. The crystal was placed in the cold stream of an Oxford Cryosystems Cobra open-flow nitrogen cryostat (Cosier & Glazer, 1986) operating at 100.0 (1) k.
Geometry. All esds (except the esd in the dihedral angle between two l.s. planes) are estimated using the full covariance matrix. The cell esds are taken into account individually in the estimation of esds in distances, angles and torsion angles; correlations between esds in cell parameters are only used when they are defined by crystal symmetry. An approximate (isotropic) treatment of cell esds is used for estimating esds involving l.s. planes.
Refinement. Refinement of F^2^ against ALL reflections. The weighted R-factor wR and goodness of fit S are based on F^2^, conventional R-factors R are based on F, with F set to zero for negative F^2^. The threshold expression of F^2^ > 2sigma(F^2^) is used only for calculating R-factors(gt) etc. and is not relevant to the choice of reflections for refinement. R-factors based on F^2^ are statistically about twice as large as those based on F, and R- factors based on ALL data will be even larger.

### Fractional atomic coordinates and isotropic or equivalent isotropic displacement parameters (Å^2^)

**Table:**

	*x*	*y*	*z*	*U*_iso_*/*U*_eq_	
N1	0.03024 (11)	0.31395 (9)	0.56765 (8)	0.0363 (2)	
N2	−0.07229 (13)	0.27732 (11)	0.70650 (10)	0.0477 (3)	
C1	0.12267 (13)	0.37351 (11)	0.52889 (10)	0.0377 (3)	
C2	0.01813 (13)	0.34187 (11)	0.67327 (10)	0.0364 (3)	
C3	0.10505 (14)	0.43720 (12)	0.74341 (10)	0.0418 (3)	
C4	0.19709 (14)	0.49686 (12)	0.70412 (11)	0.0430 (3)	
C5	0.20896 (13)	0.46594 (11)	0.59348 (10)	0.0390 (3)	
C6	0.31252 (16)	0.53099 (14)	0.55236 (13)	0.0546 (4)	
H6A	0.2979	0.5045	0.4719	0.082*	
H6B	0.4097	0.5128	0.6069	0.082*	
H6C	0.2964	0.6173	0.5519	0.082*	
O1	0.74312 (11)	0.37847 (9)	1.02381 (8)	0.0506 (3)	
O2	0.87300 (12)	0.35863 (9)	0.91200 (8)	0.0552 (3)	
N3	0.44474 (16)	0.75758 (13)	0.87306 (15)	0.0620 (4)	
C7	0.61001 (13)	0.58920 (11)	0.90248 (10)	0.0380 (3)	
C8	0.54279 (13)	0.69494 (11)	0.84017 (11)	0.0396 (3)	
C9	0.57809 (14)	0.73551 (12)	0.74452 (11)	0.0415 (3)	
C10	0.67681 (15)	0.67294 (12)	0.71301 (11)	0.0424 (3)	
C11	0.74400 (14)	0.56839 (12)	0.77538 (10)	0.0392 (3)	
C12	0.70967 (12)	0.52632 (10)	0.87065 (9)	0.0343 (3)	
C13	0.78005 (13)	0.41271 (11)	0.94066 (9)	0.0371 (3)	
H1	0.1227 (15)	0.3450 (13)	0.4509 (14)	0.049 (4)*	
H3	0.0993 (15)	0.4581 (13)	0.8200 (13)	0.048 (4)*	
H4	0.2602 (17)	0.5628 (15)	0.7561 (14)	0.061 (4)*	
H7	0.5865 (15)	0.5593 (13)	0.9703 (13)	0.046 (4)*	
H9	0.5270 (16)	0.8131 (14)	0.6969 (14)	0.056 (4)*	
H10	0.7025 (16)	0.7023 (13)	0.6453 (14)	0.053 (4)*	
H11	0.8116 (16)	0.5214 (14)	0.7520 (13)	0.050 (4)*	
H1N1	−0.0365 (17)	0.2494 (16)	0.5130 (15)	0.062 (5)*	
H1N2	−0.1357 (17)	0.2226 (15)	0.6504 (14)	0.056 (4)*	
H2N2	−0.0953 (16)	0.3031 (14)	0.7699 (15)	0.055 (4)*	
H1N3	0.4107 (18)	0.7223 (17)	0.9247 (17)	0.067 (5)*	
H2N3	0.395 (2)	0.8179 (18)	0.8245 (18)	0.077 (6)*	

### Atomic displacement parameters (Å^2^)

**Table:**

	*U*^11^	*U*^22^	*U*^33^	*U*^12^	*U*^13^	*U*^23^
N1	0.0421 (5)	0.0357 (5)	0.0327 (4)	−0.0030 (4)	0.0163 (4)	−0.0047 (4)
N2	0.0578 (7)	0.0513 (7)	0.0429 (5)	−0.0101 (6)	0.0294 (5)	−0.0079 (5)
C1	0.0405 (6)	0.0398 (6)	0.0341 (5)	−0.0004 (5)	0.0162 (5)	−0.0022 (4)
C2	0.0412 (6)	0.0359 (6)	0.0336 (5)	0.0034 (5)	0.0163 (4)	−0.0009 (4)
C3	0.0457 (7)	0.0433 (7)	0.0359 (5)	0.0016 (6)	0.0156 (5)	−0.0092 (5)
C4	0.0420 (7)	0.0386 (6)	0.0448 (6)	−0.0020 (5)	0.0132 (5)	−0.0103 (5)
C5	0.0372 (6)	0.0371 (6)	0.0421 (6)	0.0004 (5)	0.0148 (5)	−0.0003 (5)
C6	0.0516 (8)	0.0563 (9)	0.0584 (8)	−0.0131 (7)	0.0242 (6)	−0.0058 (6)
O1	0.0655 (6)	0.0505 (6)	0.0456 (5)	0.0114 (5)	0.0323 (4)	0.0126 (4)
O2	0.0757 (7)	0.0558 (6)	0.0455 (5)	0.0294 (5)	0.0362 (5)	0.0148 (4)
N3	0.0660 (9)	0.0531 (8)	0.0837 (9)	0.0201 (7)	0.0475 (8)	0.0152 (7)
C7	0.0419 (6)	0.0377 (6)	0.0380 (5)	0.0000 (5)	0.0197 (5)	0.0003 (5)
C8	0.0363 (6)	0.0370 (6)	0.0461 (6)	−0.0003 (5)	0.0169 (5)	−0.0025 (5)
C9	0.0404 (7)	0.0366 (6)	0.0441 (6)	0.0001 (5)	0.0130 (5)	0.0053 (5)
C10	0.0463 (7)	0.0442 (7)	0.0387 (5)	−0.0021 (6)	0.0187 (5)	0.0062 (5)
C11	0.0426 (7)	0.0414 (7)	0.0376 (5)	0.0025 (5)	0.0200 (5)	0.0007 (5)
C12	0.0378 (6)	0.0339 (6)	0.0308 (5)	−0.0006 (5)	0.0130 (4)	−0.0014 (4)
C13	0.0465 (7)	0.0354 (6)	0.0300 (5)	0.0033 (5)	0.0156 (4)	−0.0008 (4)

### Geometric parameters (Å, °)

**Table:**

N1—C2	1.3515 (14)	O1—C13	1.2490 (14)
N1—C1	1.3593 (16)	O2—C13	1.2642 (15)
N1—H1N1	1.018 (17)	N3—C8	1.3811 (18)
N2—C2	1.3316 (16)	N3—H1N3	0.904 (19)
N2—H1N2	0.938 (17)	N3—H2N3	0.89 (2)
N2—H2N2	0.921 (17)	C7—C12	1.3888 (17)
C1—C5	1.3607 (17)	C7—C8	1.4003 (17)
C1—H1	0.984 (15)	C7—H7	0.986 (15)
C2—C3	1.4090 (17)	C8—C9	1.3977 (18)
C3—C4	1.3605 (19)	C9—C10	1.3771 (19)
C3—H3	0.968 (14)	C9—H9	1.040 (16)
C4—C5	1.4163 (17)	C10—C11	1.3897 (18)
C4—H4	1.001 (16)	C10—H10	0.995 (15)
C5—C6	1.4974 (19)	C11—C12	1.3933 (16)
C6—H6A	0.9600	C11—H11	0.978 (15)
C6—H6B	0.9600	C12—C13	1.5126 (16)
C6—H6C	0.9600		
			
C2—N1—C1	122.40 (10)	H6B—C6—H6C	109.5
C2—N1—H1N1	118.6 (9)	C8—N3—H1N3	119.2 (11)
C1—N1—H1N1	118.9 (9)	C8—N3—H2N3	117.7 (13)
C2—N2—H1N2	118.7 (10)	H1N3—N3—H2N3	119.6 (17)
C2—N2—H2N2	120.4 (10)	C12—C7—C8	121.01 (11)
H1N2—N2—H2N2	117.6 (13)	C12—C7—H7	119.6 (8)
N1—C1—C5	122.30 (11)	C8—C7—H7	119.4 (8)
N1—C1—H1	115.2 (8)	N3—C8—C9	121.04 (12)
C5—C1—H1	122.5 (8)	N3—C8—C7	120.67 (12)
N2—C2—N1	118.85 (11)	C9—C8—C7	118.28 (12)
N2—C2—C3	123.65 (11)	C10—C9—C8	120.61 (11)
N1—C2—C3	117.48 (11)	C10—C9—H9	120.8 (9)
C4—C3—C2	119.94 (11)	C8—C9—H9	118.6 (9)
C4—C3—H3	121.1 (8)	C9—C10—C11	121.03 (12)
C2—C3—H3	119.0 (8)	C9—C10—H10	120.6 (9)
C3—C4—C5	121.83 (11)	C11—C10—H10	118.4 (9)
C3—C4—H4	119.0 (9)	C10—C11—C12	119.16 (12)
C5—C4—H4	119.1 (9)	C10—C11—H11	121.8 (8)
C1—C5—C4	116.05 (12)	C12—C11—H11	119.0 (8)
C1—C5—C6	122.69 (11)	C7—C12—C11	119.91 (11)
C4—C5—C6	121.25 (11)	C7—C12—C13	119.26 (10)
C5—C6—H6A	109.5	C11—C12—C13	120.83 (11)
C5—C6—H6B	109.5	O1—C13—O2	124.01 (11)
H6A—C6—H6B	109.5	O1—C13—C12	117.84 (11)
C5—C6—H6C	109.5	O2—C13—C12	118.15 (10)
H6A—C6—H6C	109.5		
			
C2—N1—C1—C5	−0.37 (18)	N3—C8—C9—C10	179.50 (12)
C1—N1—C2—N2	−178.35 (11)	C7—C8—C9—C10	−0.18 (18)
C1—N1—C2—C3	0.48 (17)	C8—C9—C10—C11	−0.16 (19)
N2—C2—C3—C4	178.51 (12)	C9—C10—C11—C12	0.43 (19)
N1—C2—C3—C4	−0.26 (18)	C8—C7—C12—C11	0.00 (18)
C2—C3—C4—C5	−0.1 (2)	C8—C7—C12—C13	179.81 (10)
N1—C1—C5—C4	0.01 (18)	C10—C11—C12—C7	−0.35 (18)
N1—C1—C5—C6	179.17 (12)	C10—C11—C12—C13	179.84 (11)
C3—C4—C5—C1	0.20 (19)	C7—C12—C13—O1	1.32 (17)
C3—C4—C5—C6	−178.97 (12)	C11—C12—C13—O1	−178.87 (11)
C12—C7—C8—N3	−179.42 (12)	C7—C12—C13—O2	−178.33 (10)
C12—C7—C8—C9	0.26 (18)	C11—C12—C13—O2	1.48 (17)

### Hydrogen-bond geometry (Å, °)

**Table:**

*D*—H···*A*	*D*—H	H···*A*	*D*···*A*	*D*—H···*A*
N1—H1N1···O2^i^	1.017 (17)	1.682 (17)	2.6901 (14)	170.6 (17)
N2—H1N2···O1^i^	0.939 (16)	1.886 (16)	2.8207 (15)	173.3 (14)
N2—H2N2···O2^ii^	0.920 (17)	1.947 (17)	2.8650 (16)	175.3 (16)
N3—H1N3···O1^iii^	0.903 (19)	2.18 (2)	3.027 (2)	156.0 (17)

Symmetry codes: (i) *x*−1, −*y*+1/2, *z*−1/2; (ii) *x*−1, *y*, *z*; (iii) −*x*+1, −*y*+1, −*z*+2.
